# Molecular genetic characterization reveals linear tumor evolution in a pulmonary sarcomatoid carcinomas patient with a novel *PHF20-NTRK1* fusion: a case report

**DOI:** 10.1186/s12885-019-5780-4

**Published:** 2019-06-17

**Authors:** Jianjun Ge, Bin Yao, Jia Huang, Xue Wu, Hua Bao, Qiuxiang Ou, Yang W. Shao, Jun Chen

**Affiliations:** 1 Department of Thoracic Surgery, Yinzhou People’s Hospital, Ningbo, Zhejiang China; 2 Department of Radiotherapy and Chemotherapy, Yinzhou People’s Hospital, 251 Baizhang E Rd, Jiangdong Qu, Ningbo, 315000 Zhejiang China; 3 Translational Medicine Research Institute, Geneseeq Technology Inc., Toronto, ON Canada; 4 Nanjing Geneseeq Technology Inc., Floor 18, Building B, 3-1 Xinjinhu Road, Pukou District, Nanjing, 210032 JS China; 5 School of Public Health, Nanjing Medical University, Nanjing, JS China

**Keywords:** Lung cancer, Pulmonary sarcomatoid carcinoma, Mutation profiling, Tumor evolution, *NTRK1* fusion

## Abstract

**Background:**

Pulmonary sarcomatoid carcinoma (SC) consists of both carcinomatous and sarcomatous tumors with high degree of malignancy, rapid progression, and poor prognosis. However, little is known regarding how pulmonary SC develops and progresses.

**Case presentation:**

A 66-year-old male was initially diagnosed with stage IIIa lung cancer containing both adenocarcinoma (ADC) and SC. Adjuvant chemotherapy was administrated post-surgery, however, recurrence with SC only soon followed. Mutation profiling of the patient’s microdissected ADC and SC components of the primary lesion and recurrent tumor was performed by targeted next-generation sequencing (NGS) of 416 cancer-relevant genes. Our data showed that primary SC/ADC and the recurrent SC shared multiple gene mutations including *EGFR, NF1, TP53, CDKN2B,* and *SMARCA4*, while both primary and recurrent SCs had a unique *TP53* exon 4 splicing mutation frequently observed in sarcoma. Interestingly, a novel *PHF20-NTRK1* fusion was acquired in the recurrent SC, which may be a potential driver for SC recurrence.

**Conclusions:**

The molecular genetic characteristics of tumor tissues at different stages reveals a linear tumor evolution model in this case, and support that the primary SC derived from the original lung ADC during the evolution of the tumor. We also identified a novel *PHF20-NTRK1* fusion, which may contribute to the disease recurrence, and that can be potentially targeted with NTRK1 inhibitors for treatment.

## Background

Lung cancer is the most common malignant tumor, of which 87% is non-small cell lung cancer (NSCLC) [1]. Adenocarcinoma (ADC) is the main histopathological subtype, accounting for more than 50% of lung cancers. In contrast, sarcomatoid carcinoma (SC), also named collision tumor, occurs with an extreme low incidence of 0.1–1.3% in all malignant tumors [2, 3]. It is composed of cancerous and sarcomatoid tumors, and it is still under debate whether the sarcomatoid tumor is differentiated from the cancerous tissue or both tumor types grow in adjacent areas at the same time. SC is featured with a high degree of malignancy, rapid progress, and resistance to conventional radiotherapy or chemotherapy [4]. The elucidation of how it develops and progresses will largely facilitate improving current treatment of SC. Here we report a lung cancer patient diagnosed with both invasive ADC and SC, followed by relapsed SC after surgery. Mutation profiling was performed on the patient’s primary and recurrent tumor samples for investigating tumor evolution and genetic alterations contributed to tumor development and progression.

## Case presentation

**Fig. 1:**
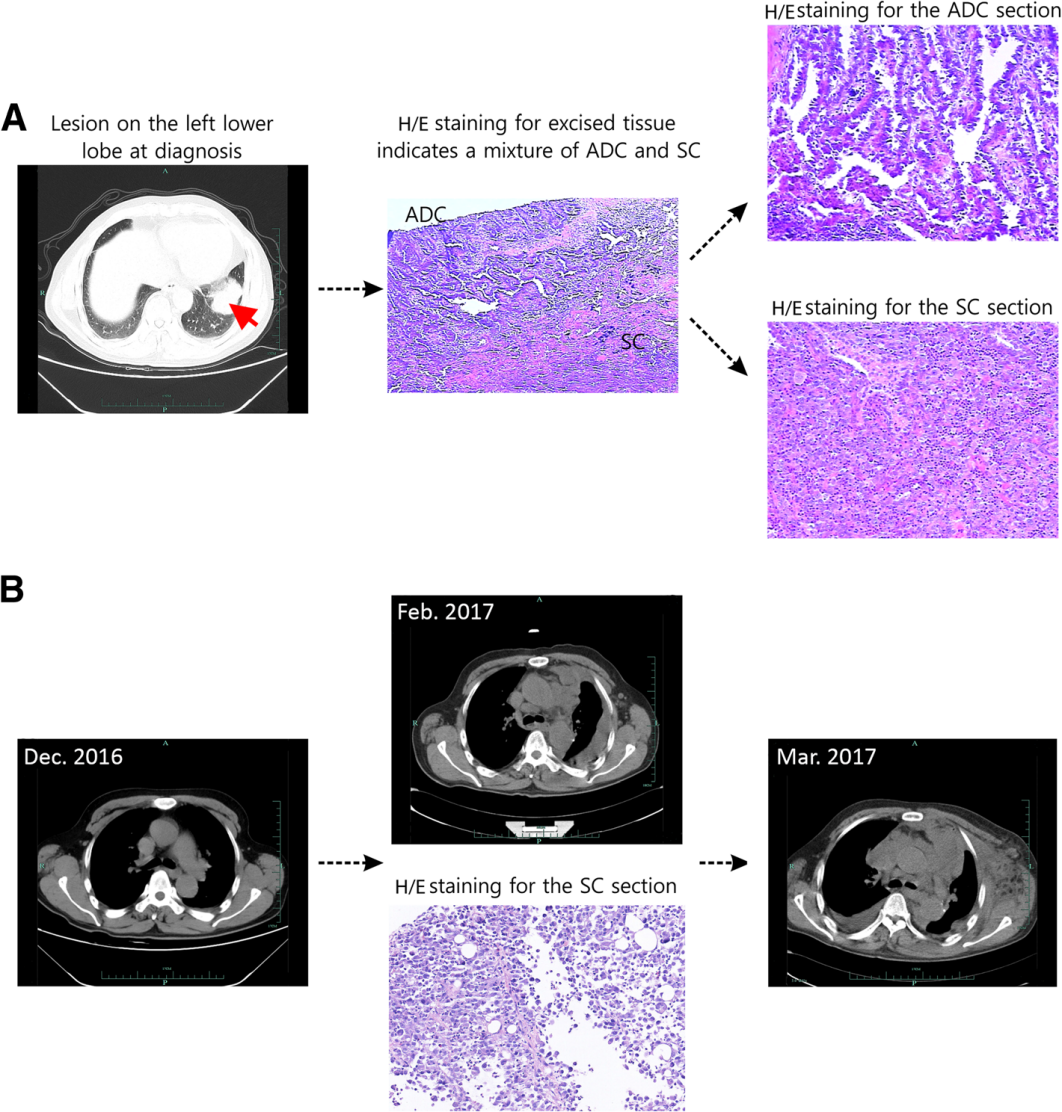
Schematic disease progression. **a** Primary tumor at diagnosis. The patient was diagnosed with lung cancer on the left lower lobe at the stage IIIa (pT2N2M0). The excised tumors from thorocoscopic lobectomy were confirmed as invasive ADC (20% of the total tumor) and SC (80% of the total tumor) morphologically, and were further sliced to separate ADC and SC sections for targeted NGS analysis. **b** Progressive increase of soft tissue masses in the anterior mediastinum, which is confirmed as SC by biopsy

A 66-year-old male with a smoking history of 30 pack-year and a drinking history of 60 g/d for 30 years visited our hospital following 1 month long coughing symptom with bloody sputum, and was diagnosed with stage IIIa (pT2N2M0) lung cancer on the left lower lobe (Fig. 1a). Thorocoscopic lobectomy was performed immediately to remove the left lower lobe of the lung and related lymph nodes. The excised tumor was confirmed as mixed invasive ADC and SC morphologically and immunohistochemically, accounting for 20 and 80% of the total tumor content, respectively (Fig. 1a).

We performed mutation profiling of the microdissected ADC and SC compartments of the surgical sample by targeting 416 cancer-relevant genes (GeneseeqOne, Nanjing Geneseeq Technology Inc., China) using hybrid capture-based targeted next-generation sequencing (NGS) on a HiSeq4000 platform (Illumina) [5]. As depicted in Table 1, we observed alterations of multiple oncogenes and tumor suppressor genes that were shared between the two compartments, including *EGFR*, *NF1*, *SMARCA4*, *and TP53 mutations*, as well as *MET* gene amplification, consistent with the prior findings that SC has a high mutation rate with the predilection for co-occurrence of more than one driver mutations [5, 8]. This may account for the high malignancy and aggressive behavior of SC and its poor response to either traditional chemotherapy or radiotherapy as seen in this patient. A rare *TP53* deletion (c.97_133 deletion) was detected in both ADC and SC tissues. This variation may result in *TP53* exon 4 mis-splicing, which is more frequently seen in sarcoma [9]. Interestingly, an additional *TP53* mutation 97-2A > T that is located right on the splicing accepter of exon 4 was only identified in SC tissue indicating a potential impact of this alteration in SC development, as well as a unique synonymous *AXIN2* mutation (Table 1). These data suggested a linear evolution model of SC progression from the ADC compartment in the primary tumor of this patient.

**Table 1:** Genetic alterations detected in the patient’s primary and recurrent tumor. The patient’s tumor samples were subject to mutation profiling by targeting a panel of 416 cancer-relevant genes including the introns of 19 genes frequently rearranged in solid tumors, including *EGFR, ALK*, and *NTRKs*. The patient’s whole blood sample was used to remove the germline mutations. Somatic mutations (filtering criteria: variant allele frequency > =2% and > =5 supporting reads from both directions) were called for each sample. Genomic fusions were identified by FACTERA [6] using default parameters. Copy number variations (CNVs) were detected using CNVkit [7] with default parameters. n.d., not detected

Genes	Alternations	Primary ADC	Primary SC	Relapsed SC
*EGFR*	p.745_750del	18.42%	13.16%	23.90%
*NF1*	p.A431S	26.03%	20.63%	33.71%
*CDKN2B*	p.1_7del	14.29%	10.00%	17.45%
*CDKN2B*	p.L34 L	5.80%	6.80%	8.98%
*SMARCA4*	Exon34–35 deletion	6.4%	9.8%	15.7%
*MET*	Amplification	2.8-fold	2.6-fold	4.8-fold
*TP53*	Exon 4, c.97_133 deletion	7.69%	7.02%	14.31%
*TP53*	Exon 4 splicing acceptor, 97-2A > T	n.d.	4.96%	10.77%
*AXIN2*	p.L128 L	n.d.	8.57%	17.05%
*PHF20-NTRK1*	Fusion	n.d.	n.d.	51.70%

**Fig. 2:**
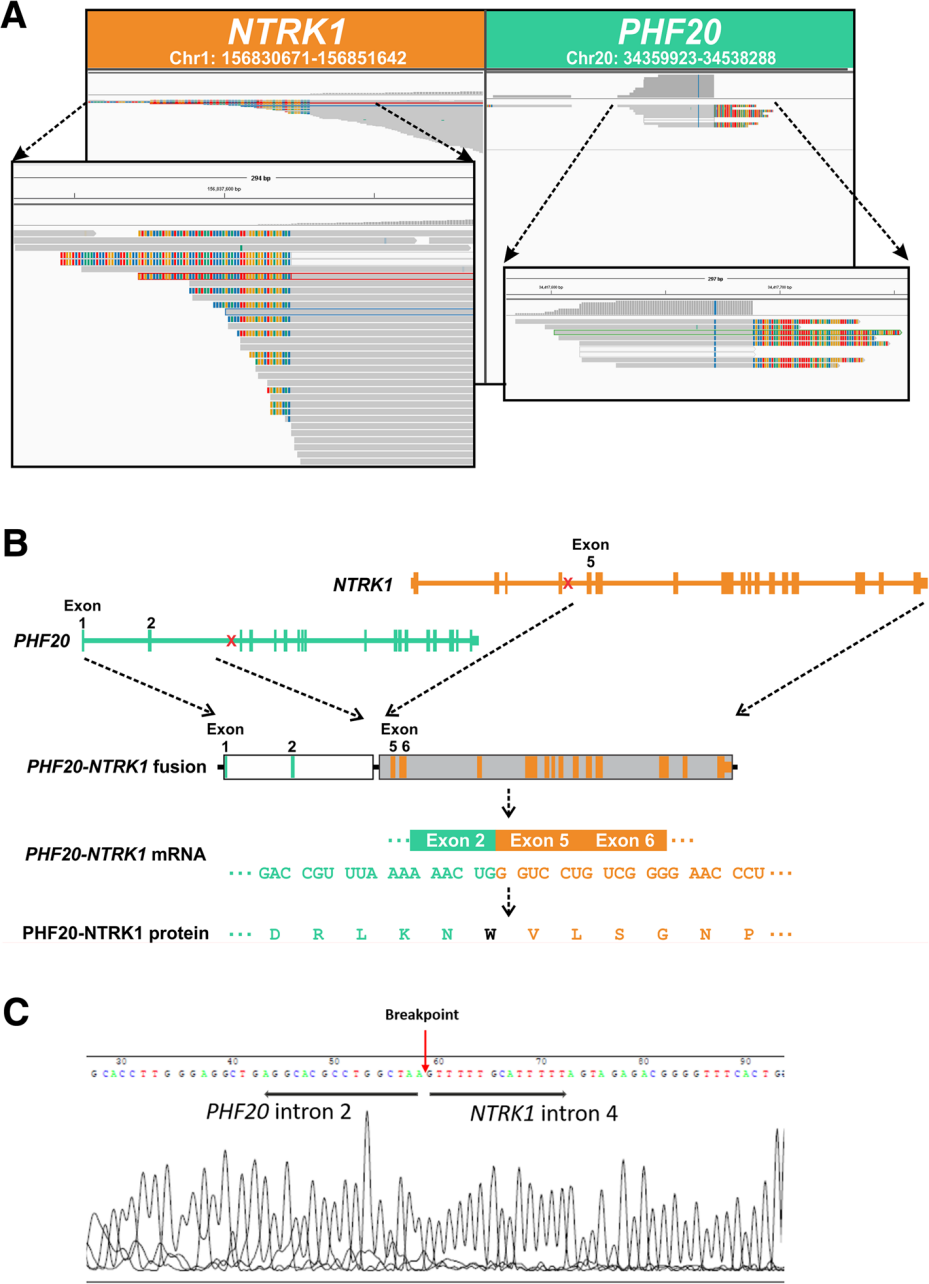
Novel *PHF20-NTRK1* fusion identified in the relapsed SC tissue. **a** *PHF20-NTRK1* fusion detected from targeted NGS only in the recurrent SC tissue. **b** A diagram depicts the *PHF20-NTRK1* fusion at the DNA level, mRNA level and protein level. **c** The confirmation of the *PHF20-NTRK1* fusion by PCR amplification of the fusion region followed by Sanger sequencing

About 4 weeks after surgery, adjuvant chemotherapy (carboplatin 0.15 D1–3 + pemetrexed 0.8 D1) was administrated to the patient. However, the patient was diagnosed with cancer relapse within a month. CT scan revealed that soft tissue masses progressively increased in the anterior mediastinum, which was further confirmed as SC by biopsy (Fig. 1b). Genetic characteristics of the recurrent SC was also performed using targeted NGS. Aside from the alterations seen in primary SC tumor tissue, the relapsed SC acquired a novel *PHF20-NTRK1* fusion where *PHF20* intron 2 fused to the intron 4 of *NTRK1* at a high variant allele frequency (VAF) (Table 1 and Fig. 2a), resulting in a *PHF20-*exon 2: *NTRK1*-exon 5 fusion mRNA with potential in-frame translation (as depicted in Fig. 2b). The resultant fusion protein preserves the whole TRKA kinase domain of NTRK1, and therefore may constitutively activate NTRK1 and contribute to the oncogenesis of the relapsed SC. We further validated the presence of this gene fusion at DNA level in the recurrent SC by PCR amplification of the fusion region followed by Sanger sequencing for sequence confirmation (Fig. 2c). Due to the presence of multiple driver gene alterations, and the unavailability of NTRK1 inhibitor, the patient then received mediastinal tumor palliative radiotherapy (DT = 18Gy/9F), but responded poorly to the treatment and deceased 16 weeks post-operation (Fig. 1b).

## Discussion and conclusions

Pulmonary SC is a rare and aggressive subset of NSCLC with limited treatment options. The understanding of the molecular traits of SC is limited due to the relative rarity of these tumors. Prior studies showed that pulmonary SC tends to have either known driver mutations or high tumor mutation burden [10], and survival probability decreased in patients with mutations detected compared with those without mutations [8]. Point mutations of *TP53* and *KRAS* were most frequently seen in PSC, and *KRAS* mutations, either alone or in combination with *TP53* aberrations, were associated with poor survival [8, 11]. *EGFR* L858R and G779C mutations have also been reported in PSC [8, 11]. In this study, *EGFR* exon19 deletion was detected in both ADC and SC, and we also observed the co-occurrence of multiple mutations of other genes including *NF1, TP53, CDKN2B*, and *SMARCA4*, which may collectively account for the poor response to standard treatments including chemotherapy and radiotherapy, thus resulting in the rapid progression of the disease. Aside from point mutations, we also observed copy number gain of *MET* in both primary and recurrent tumors, which was shown to be less frequent (1/23, 4%) in PSC [11].

Furthermore, a rare *PHF20-NTRK1* fusion was observed in the recurrent SC only. Since mutations such as *EGFR* exon19 deletion was detected across all samples at VAFs of high confidence established by NGS testing method [12, 13], it is unlikely that the absence of the *PHF20-NTRK1* fusion was attributed to the low tumor purity of primary tumor samples. Therefore, these lines of evidence corroborate that *PHF20-NTRK1* was newly acquired in the recurrent tumor during disease progression. Recently, TRK inhibitors including larotrectinib [14] and entrectinib [15], have been shown to induce clinical meaningful and durable response in patients with *NTRK* fusion-positive solid tumors. The patient could therefore potentially have benefited from those targeted therapies.

Lastly, it remains a mystery how SC coexists with other cancer subtypes, whether both develop from the same cancerous origin or one is evolved from the other. Our study showed that the patient’s SC tissue carried all the gene alterations identified in the ADC and two unique alterations including *TP53* splicing mutant and a synonymous single nucleic acid variation in *AXIN2*, suggestive of a linear evolutionary pattern from primary ADC to SC and further the relapsed SC in the patient.

In summary, mutation profiling of the patient’s primary and relapsed tumors revealed a linear tumor evolution model of PSC derived from primary ADC. We also identified a novel *PHF20-NTRK1* fusion that may contribute to SC recurrence post-surgery, and the patient could therefore potentially have benefited from targeted therapies against TRKs.

## Data Availability

The datasets generated and analyzed during the current study are not publicly available in order to protect the patient’s privacy.

## Abbreviations

ADC: Adenocarcinoma
NGS: Next-generation sequencing
NSCLC: Non-small cell lung cancer
SC: Sarcomatoid carcinoma
VAF: Variant allele fraction
